# Corrosion Behavior and Mechanical Properties of Metallic Materials

**DOI:** 10.3390/ma18051009

**Published:** 2025-02-25

**Authors:** Xiaogang Li, Xu Zhang, Xuelan Yan, Haiquan Zhang

**Affiliations:** 1 Institute of Nuclear and New Energy Technology, Tsinghua University, Beijing 100084, China; haiquanzh@mail.tsinghua.edu.cn; 2 Beijing Institute of Astronautical Systems Engineering, Beijing 100071, China; 3 Gansu Academy of Sciences, Lanzhou 730030, China; yanxuelan-01@163.com

Worldwide, more than USD 400 million/year is spent on corrosion protection and productivity losses due to corrosion. Corrosion, a process associated with chemical/electrochemical reaction, often has a deleterious consequence on mechanical properties, ultimately resulting in the degradation of a material. Metallic components, widely employed in various industries, i.e., oil, gas, marine, nuclear, fuel cells, medicine and generating electricity, often suffer from severe corrosion, which might be detrimental to service life and even cause serious accidents. Meanwhile, some extreme corrosive environments are also the main restriction on the applications of advanced metallic materials with excellent mechanical properties. Thus, understanding corrosion behavior and its effect on mechanical properties will always be of great practical significance to the development and application of metallic materials. Corrosion behavior leads to the degradation of mechanical properties, and both are affected by the service environment and the physical and chemical properties of the material itself.

The purpose of this Special Issue is to provide a research forum to report corrosion behavior and the related mechanical properties, chemical composition, and microstructure for metallic materials to address existing corrosion challenges and assist in the development of super-corrosion-resistant materials. Topics of interest include, but are not limited to, the studies mentioned above. Other relevant studies, such as hydrogen embrittlement, characterization of the corroded microstructure, the corrosion mechanisms of advanced materials, the method of surface treatment to improve corrosion resistance, the evolution mechanism of mechanical properties in corrosion environment, and the design of novel corrosion-resistant material, will also be considered, which could enhance our knowledge of corrosion protection. Research articles and reviews in this area of study are welcome.

Zhang et al. [1] contributed a review entitled “Surface-Modification Strategy to Produce Highly Anticorrosive Ti_3_C_2_T_x_ MXene-Based Polymer Composite Coatings: A Mini-Review” which reviewed Ti_3_C_2_T_x_ MXene/epoxy resin and MXene/waterborne polyurethane polymer anticorrosive composite coatings. It introduced the structure, characteristics, and main synthesis methods of MXenes and summarized the surface-modification strategies to improve the dispersion, compatibility, stability, and anti-aggregation properties of MXenes. This paper outlined the current challenges and future opportunities regarding MXene-based corrosion-resistant organic composite coatings.

Lin et al. [2] contributed a paper entitled “Molecular Dynamics Simulations of Displacement Cascades in BCC-Fe: Effects of Dislocation, Dislocation Loop and Grain Boundary” in which the interactions between displacement cascades and dislocations, dislocation loops, and grain boundaries in BCC-Fe were investigated. The atomic structural changes induced by displacement cascades were clarified to understand how irradiation damage affects dislocations, dislocation loops, and grain boundaries, revealing irradiation damage at the microscale.

Muthu et al. [3] contributed a paper entitled “Accelerated Formation of Oxide Layers on Zircaloy-4 Utilizing Air Oxidation and Comparison with Water-Corroded Oxide Layers” which examined the formation of an oxide layer on a Zircaloy-4 cladding tube by means of a combination of high-temperature water corrosion and air oxidation. Morphology and oxide phases by the above accelerated method were comparable to those formed through high-temperature water corrosion, which is of great engineering value in Canada Deuterium Uranium fuel integrity analyses.

Zurnadzhy et al. [4] contributed a paper entitled “Enhancing the Tensile Properties and Ductile-Brittle Transition Behavior of the EN S355 Grade Rolled Steel via Cost-Saving Processing Routes” which reports on the structural characterization and mechanical property evaluations of low-carbon micro-alloyed structural steel subjected to different technological processes at the final stage of steel sheet manufacturing. The work focused on a detailed comparison of the effect of different technologies on the properties of the rolled steel produced from the same melt and paid attention to the evaluation of the effect of structural factors influencing the strength of steel in regard to the variations in its ductile–brittle transition behavior.

Zhao et al. [5] contributed a paper entitled “Effect of Boron Content in LiOH Solutions on the Corrosion Behavior of Zr-Sn-Nb Alloy” in which the effects of boron (through the injection of boric acid) on improving the corrosion resistance of the Zr-Sn-Nb alloy in LiOH solutions were clarified. It was noted that B^3+^ was added into oxide films and, thus, the oxygen vacancies caused by Li^+^ aggregation were reduced, slowing down the oxidation of Zr-Sn-Nb alloys.

Cheng et al. [6] contributed a paper entitled “Investigation of Precipitation Behavior of a Novel Ni-Fe-Based Superalloy during High-Temperature Aging Treatment” which investigated the precipitation behaviors in a novel Ni-Fe-based superalloy developed for advanced ultra-supercritical coal-fired power plant applications. The abnormal coarsening behavior of precipitations in the novel alloy was noted, and its effects on hardness and crack were discussed.

Łosiewicz et al. [7] contributed a paper entitled “Effect of Artificial Saliva Modification on Corrosion Resistance of Metal Oxide Coatings on Co-Cr-Mo Dental Alloy” which prepared the single-layer TiO_2_-ZrO_2_ sol–gel coatings on the Co-Cr-Mo dental alloy using the method of dip-coating. The TiO_2_-ZrO_2_ coatings significantly reduced pitting corrosion susceptibility at physiological and acidic pH, with the 500 °C sintered coating showing better protective properties, which showed the potential of TiO_2_-ZrO_2_ coatings in enhancing the performance of Co-Cr-Mo dental alloys.

Faraji et al. [8] contributed a paper entitled “Effect of Natural Inhibitors on the Corrosion Properties of Grade 2 Titanium Alloy” which investigated the corrosion inhibition capabilities of natural extracts such as pomegranate juice, tomato and algae extract on the corrosion of grade 2 Titanium in basic solutions or in solutions containing chlorides. The use of the above compounds as natural inhibitors could be very important in the near future due to the fact that a large amount of more commonly diffused inhibitors is generally toxic and pollutant and the use of new natural compounds could be important in regard to global sustainability.

Chen et al. [9] contributed a paper entitled “Phase, Microstructure and Corrosion Behaviour of Al_0.3_FeCoNiCr_x_ High-Entropy Alloys via Cr Addition” which investigated the microstructure evolution of Al_0.3_FeCoNiCr_x_ high-entropy alloys and their short-term and long-term corrosion behaviors. The corrosion resistance of Al_0.3_FeCoNiCr_x_ increased with the increase in Cr content, exhibiting excellent corrosion resistance compared to the existing high-entropy alloys in the AlFeCoNiCr composition system.

Zhang et al. [10] contributed a paper entitled “Steel Catenary Riser Fatigue Assessment: Fracture Mechanics Approach Versus S–N Curve Method” which investigated the fatigue resistance of a full-scale Steel Catenary Riser girth weld by the Strength–Number of cycles curve method based on weld formation quality and fracture mechanic approaches, and the test results are superior to the design curve E in BS 7608. The method provided a rational estimation of full-scale girth welds, and it was noted that the short crack growth phase is crucial to improving the accuracy and that the geometries of the weld cap are the predominant factors of fatigue life.

Yang et al. [11] contributed a paper entitled “Corrosion Behavior of Copper Foil on PCB Substrates Under Atmospheric Environment in Sichuan-Tibet Region of China” which investigated the corrosion behavior of copper foil on printed circuit boards exposed for one year in a closed atmospheric environment across 22 different sites in the Sichuan-Tibet region. It was noted that copper foils in areas with a large temperature change, higher humidity and more rainfall exhibit more severe corrosion, and copper sulfides are more likely to grow in humid areas.

Sulima et al. [12] contributed a paper entitled “Effect of ZrB_2_ Content on the Properties of Copper Matrix Composite” which prepared the Cu-xZrB_2_ composites reinforced with zirconium diboride (ZrB_2_) to achieve higher hardness, compressive strength and wear resistance with lower relative densities. It should be noted that there is disproportionate decrease in the corrosion resistance of this composite with an increase in the concentration of the ceramic phase in sulfuric acid environment.

Hempel et al. [13] contributed a paper entitled “Hydrogen Diffusion in Deformed Austenitic TRIP Steel—A Study of Mathematical Prediction and Experimental Validation” which investigated the hydrogen embrittlement behavior of TRIP steel. The effects of pre-deformation on hydrogen diffusion and hydrogen embrittlement were clarified, and a good agreement between the theoretical calculations and the fracture surfaces was obtained.

Dobkowska and Kubasek [14] contributed a paper entitled “On the Possibility of the Deformation of Mg and Alloys Without Preheating of Initial Billets: Understanding Their Corrosion Performance via Electrochemical Tests” which focused on the possibility of KoBo extrusion of hcp-structured Mg alloys with different chemical compositions; it also investigated the influence of major microstructural components on the corrosion behavior of Mg and its alloys. A comparison of various groups of Mg that were extruded using KoBo was created to ascertain the most corrosion resistive alloys.

Charazińska et al. [15] contributed a paper entitled “Reconstruction of the Passive Layer of AISI 304 and 316 Steel After Scratching” which investigated the reconstruction of the passive film of austenitic stainless steels after scratching. A new approach in the conducted research was to scratch the test samples under controlled conditions and then evaluate the dynamics of the passive layer reconstruction using an atomic force microscope.

Liu et al. [16] contributed a paper entitled “Numerical Simulation of Galvanic Corrosion and Electrical Insulation for TC4/304 Galvanic Couple” which investigated the galvanic corrosion and electrical insulation between TC4 Ti-alloy and 304 stainless steel coupled in pipe joints. A finite element model was utilized to examine the influence of geometric and environmental factors on the galvanic corrosion process, which showed that electrical insulation performance significantly affects the corrosion process.

Feng et al. [17] contributed a paper entitled “The Evolution of the Tensile Properties of MoS_2_-Coated Titanium Alloy Bolts Under the Synergistic Damage of NaCl Corrosion and Preloading” in which titanium alloy bolts coated with MoS_2_ (TC4+MoS_2_) and bolts treated with a composite treatment of anodizing oxidation and MoS_2_ coating (TC4+AO+MoS_2_) were corroded while the tensile properties of them were evaluated. The coatings could provide excellent corrosion resistance, and the tensile strength and fracture behavior of titanium alloy bolts after coating were slightly affected under the synergistic damage of sea water corrosion and preloading.

Donnerbauer et al. [18] contributed a paper entitled “Methodology for Hydrogen-Assisted Fatigue Testing Using In Situ Cathodic Charging” in which a setup was developed utilizing a customized electrochemical charging cell built into a dynamic testing system. The methodology for hydrogen-assisted fatigue testing was achieved using in situ cathodic charging, which could represent hydrogen charging under superimposed mechanical loading.

Gu et al. [19] contributed a paper entitled “The Effect of Low-Temperature Short-Term Annealing on the Microstructure and Properties of Ultrafine-Grained Pure Titanium, in which industrial pure titanium was processed by equal-channel angular pressing and short-term annealed to achieve better mechanical properties for industrial applications. The strengthening of industrial pure titanium after the above processes was likely due to the improved basal texture and the refined grain size.

Vertaľ et al. [20] contributed a paper entitled “Improving Biodegradable Mg-Zn(-Ca) Alloys by Surface Treatment via Plasma Electrolytic Oxidation” which investigated the influence of plasma electrolytic oxidation preparation time on the degradation resistance of Mg-1Zn and Mg-1Zn-0.4Ca alloys. The paper noted that low-alloyed, biocompatible Mg-Zn(-Ca) alloys could achieve corrosion resistance comparable to high-performance Mg-4Y-3RE-0.4Zr alloys through appropriate surface treatment.

This Special Issue highlights the novel approach and view, which is committed to clarifying corrosion behavior and the mechanical properties of metallic materials. We sincerely thank the authors who submitted manuscript to our Special Issue and also extend our sincere gratitude to the readers. We hope that our Special Issue will serve as a platform for dialog between scientists in the field of metallic materials. In addition, in view of our continued intake of high-quality articles on the topic, we have begun work on the Special Issue “Corrosion Behavior and Mechanical Properties of Metallic Materials (Second Edition)”, and submissions are now welcome. The submission address is https://www.mdpi.com/journal/materials/special_issues/GOO9WH0316 (accessed on 20 February 2025). The topics of interest include, but are not limited to, corrosion behavior, mechanical properties, chemical composition, microstructures, hydrogen embrittlement, characterization of the corroded microstructure, corrosion mechanism of advanced materials, method of surface treatment to improve corrosion resistance, the evolution mechanism of mechanical properties in corrosion environments, and the design of novel corrosion-resistant material. Research articles and reviews regarding this area of study are welcome.
